# Supplementary figures and images for: Nrg1 regulates cortical wiring and motor recovery upon traumatic injury
Source: Front Cell Neurosci. 2026 Feb 4;20:1734342. doi: 10.3389/fncel.2026.1734342 (PMC12914564; doi:10.3389/fncel.2026.1734342)

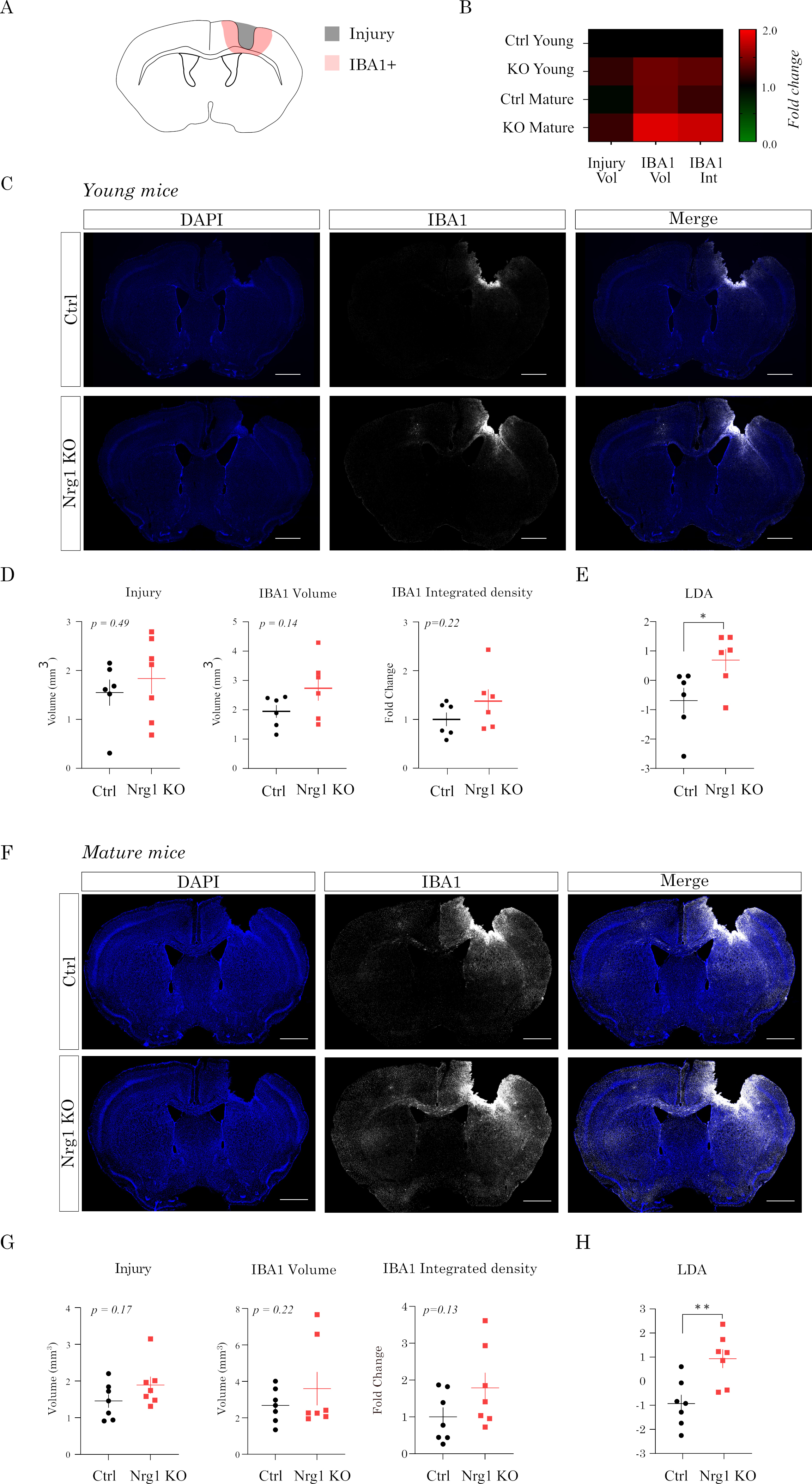

Supplement: Supplementary Figure 1 — Inflammatory response to controlled cortical damage (CCD) in neuregulin-1 (Nrg1)-deficient mice. (A) Experimental schema for lesion and inflammation quantification in young (3–4 months) and mature (9–13 months) cohorts, outlining CCD, tissue processing, region of interest (ROI) definition (perilesional cortex and distal reference), and extracted metrics (injury volume, IBA1+ volume, IBA1 integrated density). (B) Heatmap of injury and inflammatory metrics per case, grouped by genotype and age; colors indicate relative increases or decreases relative to the control young group. (C) Representative IBA1 immunostaining in perilesional cortex of young mice. IBA1+ microglia/macrophages are shown in gray, 4′,6-diamidino-2-phenylindole (DAPI) in blue. Scale Bar = 500 μm. (D) Univariate quantification of injury volume, IBA1+ volume, and ionized calcium-binding adaptor molecule 1 (IBA1) integrated density in young mice. Graphs show mean ± SEM (Ctrl, n = 6 animals; Nrg1 KO, n = 7 animals). Statistical analysis: unpaired Student’s t-test (p-values in graphs). (E) Multivariate linear discriminant analysis (LDA) integrating the same variables as in panel (D) suggests partial genotype-dependent separation in young mice (two-sided Welch t-test. *p < 0.05). (F) Representative IBA1 immunostaining in perilesional cortex of mature mice. IBA1+ microglia/macrophages are shown in gray, DAPI in blue. (G) Univariate quantification of injury volume, IBA1+ volume, and IBA1 integrated density in mature mice. Graphs show mean ± SEM (Ctrl, n = 7 animals; Nrg1 KO, n = 7 animals). Statistical analysis: unpaired Student’s t-test (p-values in graphs). (H) Multivariate linear discriminant analysis (LDA) integrating the same variables as in panel (G) shows clear genotype-dependent separation in mature mice (two-sided Welch t-test. **p < 0.01). [file Image_1.jpeg]
